# In-Depth Analysis of Diet Diary and Urine pH Measurements Improved Food Diet Reporting in Postmenopausal Women with RUTI

**DOI:** 10.1089/whr.2024.0015

**Published:** 2024-04-26

**Authors:** Juliann W. Chavez, Alana L. Christie, Philippe E. Zimmern

**Affiliations:** ^1^ Healthy Lifestyles, Healthy Futures, Knoxville, Tennessee, USA.; ^2^ Simmons Comprehensive Cancer Center Biostatistics, University of Texas Southwestern Medical Center, Dallas, Texas, USA.; ^3^ Department of Urology, University of Texas Southwestern Medical Center, Dallas, Texas, USA.

**Keywords:** food diet, women’s health, recurrent urinary tract infections

## Abstract

**Introduction:**

We proceeded with an in-depth review of diet diaries for nutrient analysis from a cohort of women suffering from uncomplicated recurrent urinary tract infection (RUTI) to validate the accuracy of our current food diet record (FDR) form and evaluate possible domains of improvement.

**Materials and Methods:**

As part of an IRB-approved study, this previously published cohort of NHANES-comparable women was analyzed for consistency of the nutrient intake over 3 days, the influence of the time between meals and urine pH, the effect of nutrient intake over interval time between meals, and seasonal and before/during the COVID-19 pandemic changes. Intrarater reliability for nutrient analysis and intrapatient variability for urine pH were computed to test for consistency.

**Results:**

Intrarater reliability for diet analysis was 91% accurate for foods and beverage matching and nutrient analysis. Mean standard deviation of urine pH readings within study participants was 0.4 (95% CI: 0.4, 0.5). An association was noted between total calories and fat consumed at breakfast and an increase in time to lunch. Calories consumed were unaffected across seasons or during the COVID-19 pandemic. Water intake during summer was significantly lower than that during fall and winter (both, *p* < 0.001). The patients who reported drinking water had a significantly lower average urine pH than women who did not report drinking water (5.8 vs. 6.2; *p* = 0.026).

**Conclusion:**

In this cohort of postmenopausal women with RUTIs, in-depth analysis of our current FDR findings led to several actionable items, which will improve our current food diet self-reporting process by our patients.

## Introduction

Self-reported diaries are often included in methods of research as well as in clinical settings. These reports allow individuals to record real-time data that provide accurate accounts of daily habits in their home setting. Information from these reports can assist clinicians to gather information and make changes to an individual’s care plan, especially for patients with diabetes, or to guide behavioral intervention therapies. These self-reports can include food diet diaries, blood sugar records and logs, sleep patterns, and medication usage.^1,2^

The food diet record (FDR) is a common tool used to capture an individual’s daily intake and nutrient consumption and is often compared with recommended dietary reference intakes to assess suboptimal intake or excessive intake of various nutrients for specific populations.^2^ FDRs that contain weekdays and weekend days as well as nonconsecutive days are often preferred as they capture a more accurate representation of usual intake patterns.^3–5^

Self-reported information may include errors. Individuals may forget to document and rely on memory or may assume that their researcher or clinician will know their daily habits based on prior questionnaires or visits. Thus, the clarification steps provided for patients filling out an FDR is crucial to ensure the most accurate data are obtained.^2,6^

Because a full 7-day collection period can be demanding on a participant, it has been found that 3- and 4-day FDRs that are unweighted are preferred and provide a more accurate record of intake, as individuals often report less intake as time extends.^2,3,6^ Furthermore, for data validity and reliability, it is important to test the reliability of the dietitian/researcher interpreting the FDR to ensure valid results, and to verify the intrapatient reporting for any errors.^7,8^

In a prior publication, we studied the relationship between urine pH and food intake, as recorded in FDRs, with the goal of possibly being able to modulate the urine pH toward a more acidic level, which hampers bacterial growth.^9–11^ We observed a similar level of information on urine pH from a 3-day diary as compared with 7 days or longer.^12^ We also noted urine pH fell into two categories, namely, always at or <6, or >6 (including patients with variable urine pH measurements).^13,14^ We also reported our analysis of d-Mannose in these diaries with the hope of finding d-Mannose-containing foods that would be best suited for lowering the pH^13^. During this process, we verified that mean nutrient intake in our study population was comparable with what was observed for a comparable age and gender group in a national food survey from NHANES, What We Eat in America (USDA).^13,15^

In this work, we performed a secondary analysis of our large dataset of postmenopausal women suffering from recurrent urinary tract infections (RUTIs) to explore additional clinical questions related to food diary and urine pH. These participants were interested in diet adjustments to consider supplements, such as d-Mannose or Hiprex™, to combat their RUTIs. Our primary goal was to explore other FDR domains, such as the intrarater reliability of the FDR analysis, missing data, days of week when the diary was recorded by patients, fluid intake based on seasons, effects of the COVID-19 pandemic, and time between urine pH measurement and the following meal. We hypothesized that this in-depth review would provide insight into whether the FDR format we used was appropriate for new patient enrollment, or if additional instructions or clarifications were needed to optimize the form used for food intake and urine pH measurements.

## Materials and Methods

The following methods (excepting the statistical methods) were detailed in our prior publication,^13^ and are provided in this study, as they remained unchanged.

### Study population

Following IRB approval (STU 042018-070), postmenopausal women (>55 years old) with a documented history of uncomplicated RUTIs (≥2 symptomatic infections within 6 months or ≥3 within 12 months along with a urine culture containing >10^5^ colony-forming units/mL)^16^ and a history of undergoing electrofulguration for antibiotic-recalcitrant RUTIs^17^ were enrolled in this study. Participants originated from two cohorts: (1) patients who were involved in a prior published study^12^ and (2) additional patients more recently enrolled. All recruited and consented participants in this study were under the care of a single FPMRS (female pelvic medicine and reconstructive surgery) physician practicing at a tertiary care urology clinic. Exclusion criteria included non-English-speaking patients, uncontrolled diabetes, chronic renal insufficiency (GFR < 30 mL/minute), stone-management specific diet, weight-loss regimen, pelvic organ prolapse stage ≥2, postvoid residual volume >100 mL, the use of chronic indwelling catheters or chronic intermittent catheterization, symptomatic UTI requiring antibiotic therapy, or if urine pH records were incomplete.

### FDR and urine pH

Participants were given preformatted charts to record urine pH using 11-panel QTEST urinalysis reagent strips (Medimpex United Inc, Bensalem, PA) at each collection time as well as their food, beverage, and dietary supplement consumption (food diary) on the same days urine pH measurements were obtained. Urine pH dipstick measurements occurred before each meal and before going to sleep. Verbal and written instructions on how to keep a food diary were given along with two examples of how to fill out the food diary chart. Verbal instructions suggest the patients to follow their normal diet, so that accurate readings can be obtained.

### Dietary intake and analysis

A registered dietitian (RD) not involved in patient care analyzed macro- and micronutrients reported from food diaries using the well-established Axxya Systems Nutritionist Pro™ software Version 7.8.0 (Axxya Systems, LLC Redmond WA) independently licensed by the analyzer, to compute the energy and nutrient composition of all foods, beverages, and supplements. Documented food diary information was manually entered into the software system, and the database reported nutrient information referenced either from the United States Department of Agriculture (USDA) database or from reported nutrient information from the food product brand as each is available in the software. When information from both sources was available, the USDA database was preferred. The same 17 nutrients plus ash, fluid intake, and weight that were studied in our previous publication were examined in this report.^13^ The nutrients selected for analysis were based on main nutrients that are included on the food label (calories, protein, carbohydrates, fat, sugar, sodium, fiber, potassium, iron, vitamin D, calcium), other nutrients found associated with changes in urine pH or change in pH (ash, fluid intake, beta-carotene), and other nutrients vitamin A, vitamin C, thiamin, riboflavin, and niacin that were of interest.

### Statistical methods

Completeness of FDRs was calculated as the percent of the meals across all patients and the percent of the urine pH measurements that should have been reported. To determine intrarater reliability, the RD analyzed 10 of the FDRs a second time, >1 year after the first analysis. The percent of foods that were exact matches, approximate matches, and foods that were unique to one analysis were each calculated. To determine the intrapatient variability in urine pH, we calculated the standard deviation of the 12 measurements for each patient, and then calculated the mean of these standard deviations across all patients. The median of the standard deviations was used for calculating intrapatient variability in nutrients consumed at the three recorded breakfasts, the meal we chose to focus on due to consistency of intake. The mean interval time in minutes with standard deviation and 95% CI between each meal (breakfast, lunch, dinner) and urine pH measurement by dipstick were also calculated. Additionally, we used mixed model analysis to compare the 17 nutrients plus ash, fluid content, and weight at breakfast to the change in urine pH from breakfast to lunch.

To determine whether we had a predominance of weekdays or weekend days recorded, we broke down the 3-day interval into 3 weekdays, 2 weekdays + 1 weekend day, or 1 weekday + 2 weekend days. We then used a mixed-model analysis to assess whether caloric intake differed between weekday and weekend day.

Similarly, we sought to assess for any seasonal effects on the FDRs using mixed-model analyses to compare caloric intake and total moisture intake by season. Further investigation for patients who did not record any water intake (tap water, mineral water, *etc.*) was evaluated to determine if total moisture (foods and beverages) was different in women who reported water intake to those women who did not record drinking water. As a further attempt to determine if the lack of reporting water was missing data or simply a lack of drinking water, we tested for a difference in urine pH between the two groups. To assess for an effect of the COVID pandemic, we tested for differences in the 17 nutrients plus ash, fluid content, and weight between the year 2019 (*n* = 22) and years 2020–2021 (*n* = 21).

All analyses were completed at the 0.05 significance level without adjustment for multiple comparisons using SAS 9.4 (SAS Institute Inc., Cary NC).

## Results

### Patient demographics

From February 2019 to April 2021, 43 women had met study criteria. These 43 women supplied 507 urine pH measurements and diet data from 378 meals. Patient characteristics are shown in Supplementary Table S1. Patients had a median age of 71 (IQR 66–75) years and were mostly Caucasian (89%).

### Source of missing data

Individual reports of meals and pH measurements were recorded for 97% patients. Four patients did not record all meals (*n* = 9 meals were not recorded). Two reported that they skipped the meal (*n* = 6 meals), and the other two (*n* = 3 meals) did not have any comment on skipping versus forgetting to record the meal.

### Intrarater reliability

Food matching was calculated for intrarater reliability. On repeat FDR analysis, 229 foods matched exactly by name, and 153 foods were approximate matches. There were 19 instances where foods had approximate matches due to being broken down to individual parts in one analysis. Forty-four foods were unique to one analysis, for an overall 91% food match rate. In the 229 exact food matches, 155 (68%) also matched exactly on portion size. There were 16 foods (7%) that were reported in different measurements (*e.g.,* 1 tablespoon vs. 1 teaspoon), and 58 foods (25%) used the same measurement unit, but with different amounts (*e.g.,* 0.5 cups vs. 1 cup). These 74 foods had median absolute difference in measured weight of 37.8 grams (IQR: 15.3–100) between the two reads.

### Intrapatient variability

**FIG. 1.**
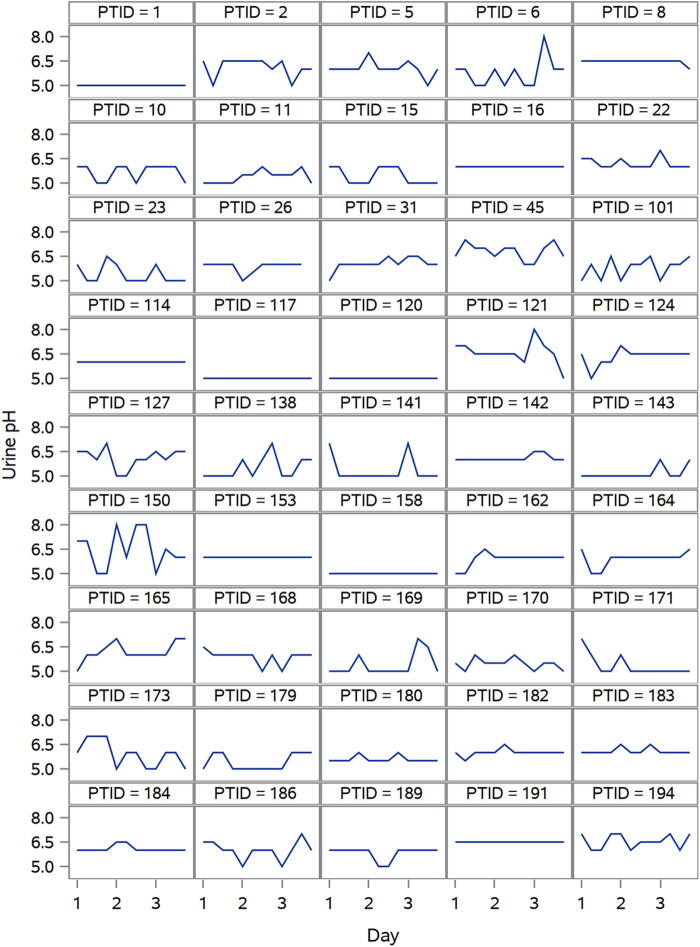
Within-patient variation in urine pH over the 3-day study period.

Within-patient variations in urine pH are shown in Fig. 1. After taking the standard deviation of urine pH values for each patient, we found that the mean standard deviation was 0.4 (95% CI: 0.4, 0.5). We did not observe meaningful variation in any of the nutrients (Table 1).

**Table 1.** Intrapatient Variability in Nutrients Consumed at Breakfast

	Median standard deviation (IQR)
Weight (g)	192.9 (67.4–266.6)
Kcal	76.2 (36.6–132)
Protein (g)	4.3 (2.1–7.5)
Carbohydrates (g)	11.9 (4.6–17.2)
Fat (g)	5 (1.7–7.5)
Sugar (g)	5.8 (1.7–11.3)
Dietary fiber (g)	1.4 (0.5–2.6)
Vitamin A (RE)	47.1 (8.8–81.5)
Vitamin C (mg)	4.1 (0.2–14.4)
Vitamin D (mcg)	0.8 (0–1.5)
Sodium (mg)	119.8 (52.2–214.4)
Potassium (mg)	158.2 (82–245)
Calcium (mg)	53.6 (18.9–153.8)
Iron (mg)	1.1 (0.4–2)
Beta carotene (mcg)	14.7 (1.8–27)
Thiamin (mg)	0.1 (0.1–0.2)
Riboflavin (mg)	0.2 (0.1–0.3)
Niacin (mg)	2 (0.6–2.8)
Fluid (g)	173.5 (53.8–272.2)
Ash (g)	0.6 (0.3–1.2)

### Daily timing

Timing between urine pH measurements and food or beverage intake is shown in Table 2. The earliest time (in minutes) between meal and pH measurements was 22 min, and the longest time was 286 min. Urine collection typically occurred a short time before meals, with the longest being before breakfast. We did not see a significant association between urine pH and the time between urine pH and meals.

**Table 2.** Mean Time in Minutes between Urine pH Measurements and Meals (*n* = 43)

	Mean (95% CI)	Standard error
Morning urination to breakfast	48 (33, 64)	7.6
Breakfast to prelunch urination	267 (249, 285)	8.8
Prelunch urination to lunch	22 (14, 31)	4.1
Lunch to predinner urination	286 (270, 302)	7.9
Predinner urination to dinner	34 (22, 47)	6.0
Dinner to nighttime urination	221 (204, 238)	8.5

**FIG. 2.**
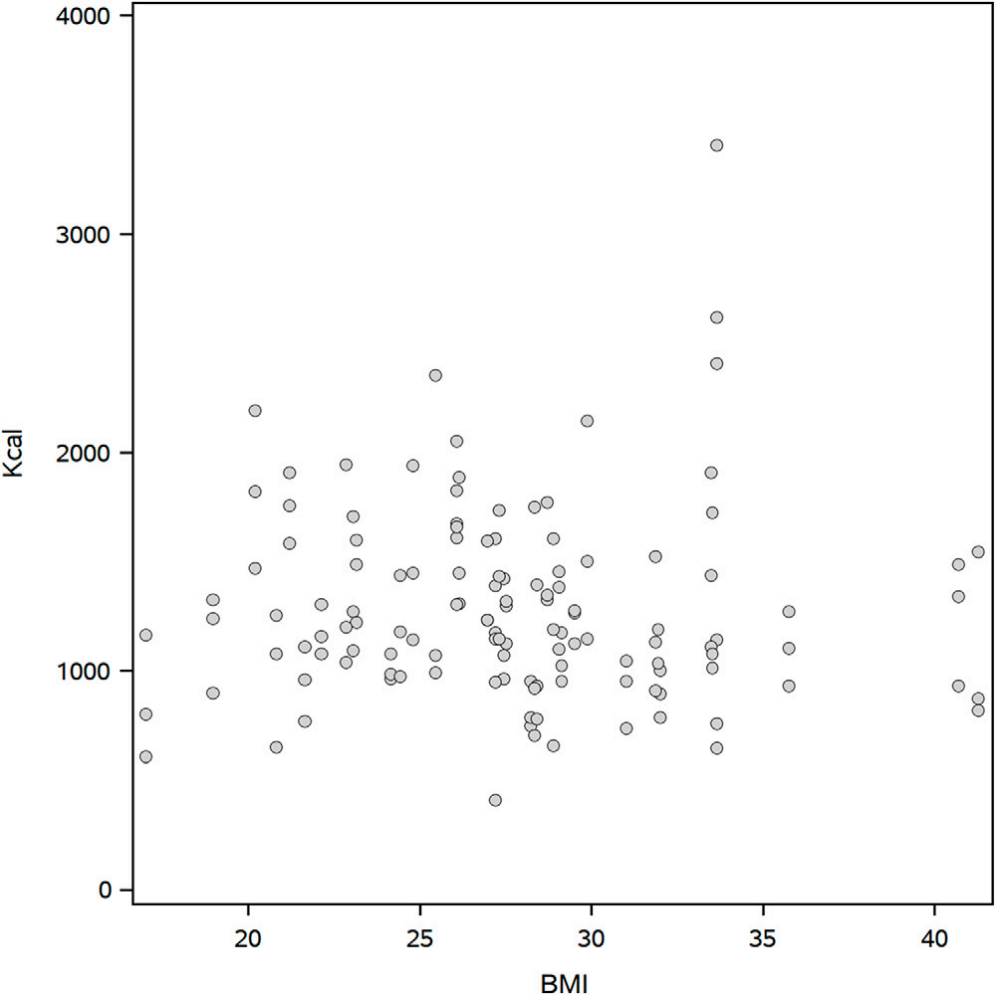
Scatterplot of patient BMI and their daily caloric intake. BMI, body–mass index.

We examined the association of the time between breakfast and lunch and the nutrients that were consumed at breakfast (Table 3). Every additional calorie eaten at breakfast was associated with a significantly increased interval between breakfast and lunch by 0.11 min. Weight of the meal was also significantly associated with increased time between meals, as well as protein, carbohydrates, sugar, vitamin A, sodium, and potassium. There was no association with moisture intake at breakfast and time between meals. Additionally, no association was observed between body mass index (BMI) and caloric intake (Fig. 2).

**Table 3.** Mixed Model Estimates for Nutrients at Breakfast and Minutes between Breakfast and Lunch

	Estimate (95% CI)	*p*
Weight (g)	0.06 (0.01, 0.11)	0.033
Kcal	0.11 (0.01, 0.21)	0.036
Protein (g)	1.95 (0.29, 3.62)	0.022
Carbohydrates (g)	0.67 (0.01, 1.32)	0.047
Fat (g)	0.43 (−1.36, 2.23)	0.6
Sugar (g)	0.13 (0.00, 0.27)	0.046
Dietary fiber (g)	2.62 (−2.45, 7.68)	0.3
Vitamin A (RE)	0.08 (0.03, 0.13)	0.0010
Vitamin C (mg)	−0.01 (−0.04, 0.01)	0.4
Vitamin D (mcg)	−0.00 (−0.00, 0.00)	0.8
Sodium (mg)	0.07 (0.01, 0.13)	0.017
Potassium (mg)	0.07 (0.01, 0.12)	0.019
Calcium (mg)	−0.00 (−0.01, 0.01)	0.9
Iron (mg)	2.27 (−0.97, 5.51)	0.17
Beta carotene (mcg)	0.01 (−0.01, 0.03)	0.3
Thiamin (mg)	−0.12 (−1.79, 1.54)	0.9
Riboflavin (mg)	0.01 (−1.66, 1.68)	0.9
Niacin (mg)	0.23 (−0.28, 0.75)	0.4
Fluid (g)	0.04 (−0.02, 0.09)	0.16
Ash (g)	0.18 (−1.81, 2.18)	0.86

### Weekly timing

No significant difference in caloric intake was noted by weekday versus weekend. Percentage of weekend and weekdays were: 25/43 (58%) 3 weekdays, 6/43 (14%) 2 weekdays and 1 weekend day, and 12/43 (23%) 1 weekday and 2 weekend days.

### Seasonal timing

Across seasons, calories were similar (Table 4; *p* = 0.5). There was a significant association between moisture and season: summer was significantly lower than fall (*p* = 0.0002) and winter (*p* = 0.0093). Nine patients did not report drinking any water or any other sources of fluid intake. The daily total moisture intake (fluids in both foods and beverages) in women who reported drinking water was 1675 g versus 739 g in women who did not report drinking water (*p* = 0.0001). The patients who reported drinking water had a significantly lower average urine pH than the women who did not report drinking water (5.8 vs 6.2; *p* = 0.026). Five of nine (56%) patients who did not report drinking water were in the study over the summer, compared with 9/34 (26%) patients who did report drinking water.

**Table 4.** Daily Intake by Season

	Daily moisture intake (g)		Daily caloric intake	
	Mean estimate (95% CI)	*p*	Mean estimate (95% CI)	*p*
Winter (Dec–Feb)	1687 (1258, 2116)	0.0020	1263 (1011, 1515)	0.5
Spring (Mar–May)	1467 (1008, 1925)		1213 (941, 1486)	
Summer (Jun–Aug)	960 (635, 1284)		1220 (1029, 1411)	
Fall (Sep–Nov)	1881 (1557, 2205)		1399 (1208, 1590)	

### Impact of the COVID-19 pandemic

We found no significant differences in caloric intake or nutrient intake based on enrollment in 2019 or 2020–2021, nor were there any difference in BMI between these two study periods. However, a significant increase in moisture intake was observed (Table 5; 1062 g in 2019 compared with 1875 g in 2020–2021; *p* < 0.0001). This association was independent of the season.

**Table 5.** Mixed Model Estimates for Total Nutrient Intake in pre-COVID (2019) versus during COVID Pandemic (2020–2021)

	Mean estimate (95% CI)		
	Pre-COVID-19 (2019)	During COVID-19 (2020–2021)	*p*
Weight (g)	1582 (1300, 1865)	2320 (2044, 2596)	0.0005
Kcal	1229 (1052, 1406)	1409 (1236, 1582)	0.15
Protein (g)	57.0 (49.5, 64.4)	60.4 (53.1, 67.7)	0.5
Carbohydrates (g)	137.4 (113.5, 161.3)	155.3 (132.0, 178.6)	0.3
Fat (g)	51.6 (41.9, 61.2)	59.5 (50.1, 69.0)	0.2
Sugar (g)	87.6 (29.6, 145.7)	61.4 (4.7, 118.1)	0.5
Dietary fiber (g)	14.0 (11.5, 16.5)	14.3 (11.9, 16.8)	0.8
Vitamin A (RE)	818.3 (465.1, 1171)	628.6 (283.6, 973.7)	0.4
Vitamin C (mg)	1665 (0.0, 3542)	73.3 (0.0, 1907)	0.2
Vitamin D (mcg)	4118 (0.0, 12323)	3929 (0.0, 11945)	0.9
Sodium (mg)	1900 (1571, 2230)	2046 (1724, 2368)	0.5
Potassium (mg)	1613 (1377, 1848)	1786 (1556, 2016)	0.3
Calcium (mg)	1237 (487.0, 1986)	1001 (269.1, 1734)	0.7
Iron (mg)	11.1 (8.0, 14.1)	10.6 (7.6, 13.6)	0.8
Beta carotene (mcg)	1842 (830.5, 2854)	2229 (1241, 3217)	0.6
Thiamin (mg)	6.7 (0.0, 15.7)	3.2 (0.0, 12.0)	0.6
Riboflavin (mg)	7.6 (0.0, 16.6)	3.5 (0.0, 12.3)	0.5
Niacin (mg)	26.9 (13.3, 40.5)	16.2 (2.9, 29.5)	0.3
Fluid (g)	1062 (810.6, 1313)	1875 (1629, 2120)	<0.0001
Ash (g)	7.9 (5.6, 10.2)	7.2 (4.5, 10.0)	0.7

## Discussion

Our goal was to examine the diet diaries for nutrient analysis over 3 days in a cohort of women suffering from uncomplicated RUTIs, in whom we were interested to determine the relationship between their food intake and their urine pH, with the knowledge that a lower pH could hinder bacterial growth.^9–11^

Intrarater reliability of the FDR interpretation was very strong (91% accuracy) for food matching and identifying dietary intake, which was based on having knowledge of foods in the database with the most accurate nutrient content.^8,18–21^ However, more clarity is needed on how patients can estimate portion size, which led us to add more detailed guidelines in our instructions for patients, including graphics (Supplementary Appendix S1).

Self-reported food diaries and diet records have been found to come with errors.^2,3,6–8^ However, in this older population, 97% of our patients provided complete recording of meals, mealtimes, and urine pH measurements. Possibly, providing sample intake forms and detailed instructions in-person, with additional follow-up as needed, helped individuals feel properly instructed to complete the FDR. Although we did not observe any differences in diet based on weekday versus weekend day in this cohort, prior studies have offered more specific direction to include 2 weekdays and 1 weekend day.^3–5^ Therefore, for the sake of striving for accuracy of total consumption, we will encourage participants to provide the FDR from 2 weekdays and 1 weekend day.

Meal timing, meal size, nutrient content, and BMI have been associated with the length of time between meals or the time food is eaten.^22,23^ Higher calorie intake and weight of food often lead to a larger interval time between meals, presumably as a result of greater satiety. In this study, weight of the meal and calories were significantly associated with time between meals. No significant differences were found between nutrient intake and season, BMI and macronutrient content of diets, or pre-COVID-19 versus during the COVID-19 pandemic.

Our most intriguing outcome was that the amount of moisture (water and water in foods) consumed was reportedly higher in fall and winter as compared with spring and summer. Increased water intake has been found to reduce the number of RUTIs.^24^ In our further exploration of the amount of water and water in foods consumed, the women who reported drinking water (tap water, mineral water, tonic water, and soda water) had a significantly lower average urine pH than those who did not report drinking water (5.8 vs 6.2; *p* = 0.026). Assuming no underreporting, this observation could provide support to prior studies indicating that water consumption may be an effective approach to reduce the risk of increased RUTIs, not only by mechanical flushing out of the bacteria from the bladder but also by lowering urine pH. To avoid underreporting of fluid intake outside of regular meals, we added an example on our revised FDR form of where to record this information.

Limitations of this study include the predominantly Caucasian population managed at our tertiary care center, which can limit generalizability to other races and ethnicities. In addition, it is possible that this older population had more time availability to thoroughly complete these FDRs, resulting in low missing data and high reliability. Finally, we recognize that recording an FDR may influence the foods eaten or otherwise not be representative of their “nondiary” days; however, we already noted that a few patients occasionally commented that their intake differed from their usual diet. As a result, we added a comment section (including examples) to our revised FDR, available in Supplementary Appendix S1.

## Conclusions

Taken together, this in-depth diet analysis led to several actionable items for improving our current patient-reported FDR process. These include providing more instructions regarding estimating portion size, including all food and drink that was not consumed at regular mealtimes, recording diet for at least 1 weekday and 1 weekend day, and detailing any changes in diet or usual eating pattern(s) in a comment box. These changes in patient-reported FDRs should improve the accuracy of future diet data collected among postmenopausal women suffering from uncomplicated RUTIs.

## Supplementary Material

Supplementary Table S1

Supplementary Appendix S1

## Abbreviations Used

BMI: body mass index
CI: confidence interval
COVID: coronavirus disease
FDR: food diet record
FPMRS: female pelvic medicine and reconstructive surgery
IRB: institutional review board
IQR: interquartile range
NHANES: national health and nutrition examination survey
QTEST: brand of urinalysis reagent strip
RD: registered dietitian
RUTI: recurrent urinary tract infection
USDA: United states department of agriculture
