# Supplementary material for: In-Depth Analysis of Diet Diary and Urine pH Measurements Improved Food Diet Reporting in Postmenopausal Women with RUTI
Source: Womens Health Rep (New Rochelle). 2024 Apr 26;5(1):367–75. doi: 10.1089/whr.2024.0015 (PMC11257122; doi:10.1089/whr.2024.0015)
Supplement: Supplementary Table S1 [file whr.2024.0015_supp_tables1.pdf]

Supplemental Table 1. Patient characteristics

	**N (%)** **(n = 43)**
Median age, years (IQR)	71 (66-75)
Race	
Asian	2 (6%)
Black	2 (6%)
White	32 (89%)
Median BMI (IQR)	27.5 (24.1-31)
Median gravidity (IQR)	3 (2-3)
Median parity (IQR)	2 (2-3)
Hysterectomy	29 (67%)
Hormone replacement therapy	
None	10 (23%)
Local	30 (70%)
Systemic	3 (7%)
Sexually active	19 (53%)
Diabetic	7 (16%)
Ever smoker	12 (28%)
