# Supplementary material for: In-Depth Analysis of Diet Diary and Urine pH Measurements Improved Food Diet Reporting in Postmenopausal Women with RUTI
Source: Womens Health Rep (New Rochelle). 2024 Apr 26;5(1):367–75. doi: 10.1089/whr.2024.0015 (PMC11257122; doi:10.1089/whr.2024.0015)
Supplement: Supplementary Appendix S1 [file whr.2024.0015_suppl_datas1.pdf]

Instructions for Participants

Thank you for your participation in this study! Your contribution will help to add understanding about recurrent urinary tract infections. Below are instructions on how to record urine data using the dipsticks you were given, as well as your food and drink record as discussed in the clinic.

Please take urine measurements BEFORE eating a meal.

If you forget, please still take measurements and indicate on the record sheet that the measurement was taken after eating.

How to Read Measurements on the Urine Dipstick

1. Collect a fresh urine sample in a clean, dry container.
2. When taking out a dipstick from the bottle, try to replace the container cap quickly so that the other strips are not exposed more than necessary before use.
3. Using one dipstick, completely immerse the reagent pads of the strip in the urine sample and then remove quickly.
4. While removing the dipstick from the urine sample container, run the edge of the strip against the rim of the container to remove excess urine, then place the dipstick on a flat level surface.
5. Wait about 30 seconds, and compare your dipstick to the chart on the dipstick bottle (below).
    a. Look at the row labeled “Glu” (at the bottom of the chart), and find the color on the chart that best corresponds to the color on that section of your dipstick. Write down the number listed above that color on your chart.
    b. Next, look at the row labeled “pH” (5 rows above the “Glu” row), and find the color on the chart that best corresponds to the color on that section of your dipstick. Write down the number listed above that color on your chart.
6. Discard urine in the toilet.
7. Repeat this process for a total of four times each day for a total of 7 days.

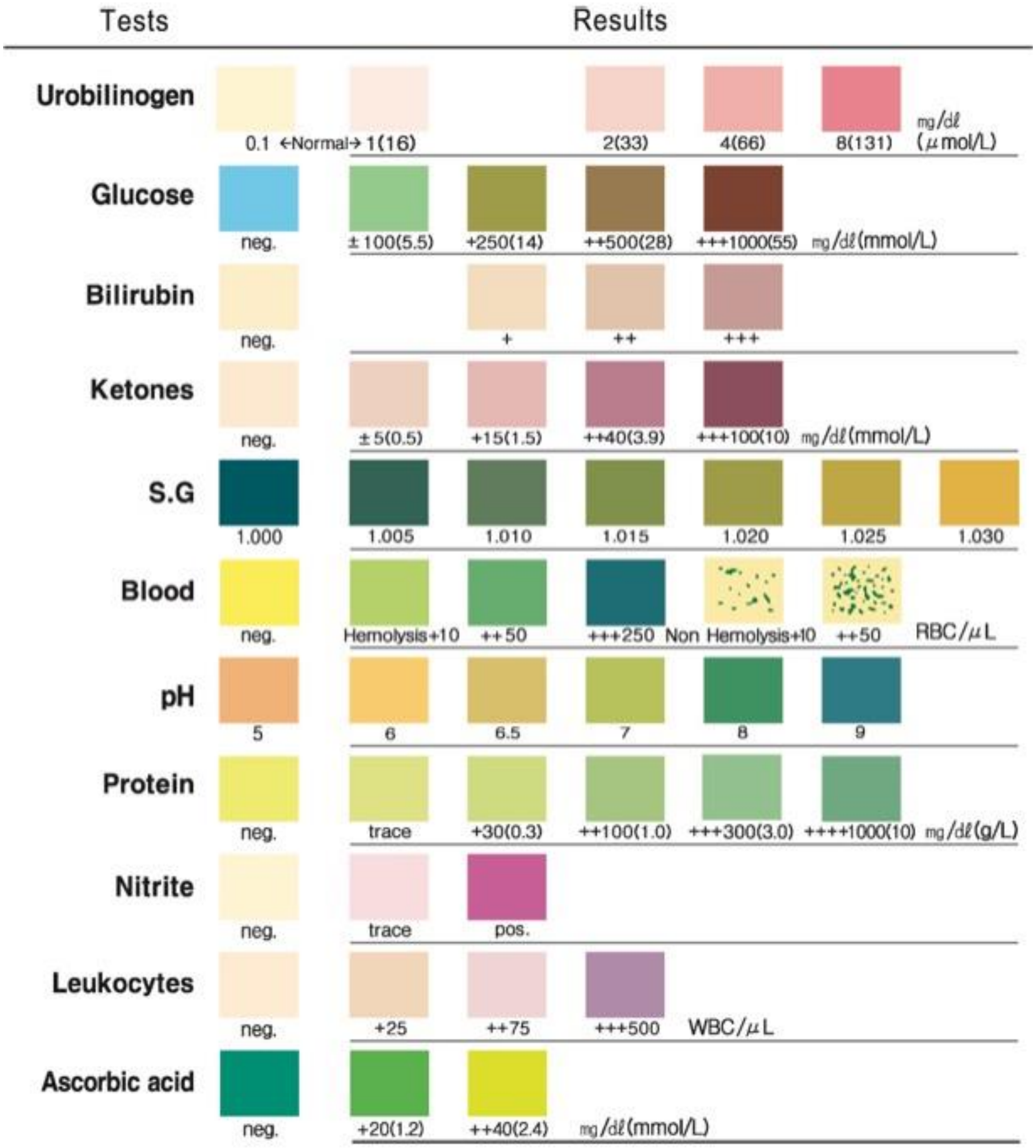
Tests
Results
Urobilinogen
0.1 ←Normal→ 1(16)
2(33)
4(66)
8(131)
mg/dℓ (μmol/L)
Glucose
neg.
±100(5.5)
+250(14)
++500(28)
+++1000(55)
mg/dℓ(mmol/L)
Bilirubin
neg.
+
++
+++
Ketones
neg.
±5(0.5)
+15(1.5)
++40(3.9)
+++100(10)
mg/dℓ(mmol/L)
S.G
1.000
1.005
1.010
1.015
1.020
1.025
1.030
Blood
neg.
Hemolysis+10
++50
+++250
Non Hemolysis+10
++50
RBC/μL
pH
5
6
6.5
7
8
9
Protein
neg.
trace
+30(0.3)
++100(1.0)
+++300(3.0)
++++1000(10)
mg/dℓ(g/L)
Nitrite
neg.
trace
pos.
Leukocytes
neg.
+25
++75
+++500
WBC/μL
Ascorbic acid
neg.
+20(1.2)
++40(2.4)
mg/dℓ(mmol/L)

How to Keep a Food Diary

1. Record each food or beverage consumed in the column labeled Food / Beverage, as well as the approximate time at which the item was consumed.
2. You may want to record **one weekday (**or **workday)** and **one Saturday** or **Sunday (**or **day off)**.
3. In the column labeled amount, estimate the amount consumed using standard units of measurements or using the serving size given on the package of the food. For example, measurements can be given in tablespoons, cups, ounces, grams, etc. Alternatively, packaged food can be described using terms such as "1 can of Campbell's tomato soup, 2 slices of bread, 1 snack size bag of Lays' chips", etc.
4. Include:
    a. How much food you ate. See the suggestions below to estimate portion sizes. If the food comes in a package, just write down the package size. Example: 175 mL container of yogurt.
    b. How the food is cooked (for example: fried, baked, boiled, barbecued).
    c. Anything you add to food, during or after cooking. Example: cream, sugar, oil, butter, jam, syrup, ketchup or other sauces, dressings or condiments.
    d. Details about restaurant foods, fast foods, or packaged foods (for example: McDonald's Big Mac® or KFC® chicken).
5. Measure the food you eat for a day or two to help you understand how much you eat and drink. Use measuring cups and spoons.

To estimate portion sizes, use the guidelines below:

3 oz (85g) of meat	a deck of cards
1 1⁄2 oz (50 g) of cheese	2 white erasers or 2 dominos
1 cup (250 mL)	a baseball or tennis ball
1⁄2 cup (125 mL)	½ a tennis ball
1 medium piece of fruit	a fist (use your hand)
2 Tbsp (30 mL)	1 golf ball
1⁄4 cup (60 mL)	2 golf balls
1 tsp (5 mL)	a thumb tip or one die

Include any vitamins or supplements taken around that same time.

6. In the comment column section include any other information.
7. Please use a new sheet for each day during the 3-day period.

Examples for Breakfast foods:

**Time**	**Food/ Beverage**	**Amount**	**Brand/Restaurant**
**8:00 am**	**Cereal**	**¾ cup**	**Cheerios**
	**Skim Milk**	**8 oz**	**Kroger**
	**Strawberries**	**1 cup**	**fresh**
	**Coffee**	**1 cup with 2 creams and 2 sugars**	
	**Egg Sandwich**	**1**	**McDonalds**
**9:30 am**	**Multivitamin**	**1**	**Nature Made**
	**Water**	**8 oz**	

Examples for Lunch/Dinner

**Time**	**Food/ Beverage**	**Amount**	**Brand/Restaurant**
**4-5:00 pm**	**Water**	**16 oz**	
**6:00 pm**	**Salad with lettuce, tomatoes, carrots**	**1 cup**	
	**Dressing**	**2 tablespoons**	**Wish-Bone Italian**
	**Spaghetti**	**2 cups**	
	**Meatballs**	**4 small**	
	**Pasta Sauce**	**¼ cup**	**Barilla Marinara**
	**Garlic Toast w/ butter**	**1 slice**	
	**Sweet Iced tea**	**12 oz**	**Lipton**
**8:00 pm**	**Ice Cream**	**½ cup**	**Blue Bell Homemade Vanilla**

Comments:
Usually drink more water.
Not feeling well.
My Birthday so splurged☺
